# Ketamine reduces the neural distinction between self- and other-produced affective touch: a randomized double-blind placebo-controlled study

**DOI:** 10.1038/s41386-024-01906-2

**Published:** 2024-06-25

**Authors:** Reinoud Kaldewaij, Paula C. Salamone, Adam Enmalm, Lars Östman, Michal Pietrzak, Hanna Karlsson, Andreas Löfberg, Emelie Gauffin, Martin Samuelsson, Sarah Gustavson, Andrea J. Capusan, Håkan Olausson, Markus Heilig, Rebecca Boehme

**Affiliations:** 1 Center for Social and Affective Neuroscience, Linköping University, Linköping, Sweden; 2 Center for Medical Image Science and Visualization, Linköping University, Linköping, Sweden

**Keywords:** Sensorimotor processing, Perception, Social neuroscience, Human behaviour

## Abstract

A coherent sense of self is crucial for social functioning and mental health. The N-methyl-D-aspartate antagonist ketamine induces short-term dissociative experiences and has therefore been used to model an altered state of self-perception. This randomized double-blind placebo-controlled cross-over study investigated the mechanisms for ketamine’s effects on the bodily sense of self in the context of affective touch. Thirty healthy participants (15 females/15 males, age 19–39) received intravenous ketamine or placebo while performing self-touch and receiving touch by someone else during functional MRI – a previously established neural measure of tactile self-other-differentiation. Afterwards, tactile detection thresholds during self- and other-touch were assessed, as well as dissociative states, interoceptive awareness, and social touch attitudes. Compared to placebo, ketamine administration elicited dissociation and reduced neural activity associated with self-other-differentiation in the right temporoparietal cortex, which was most pronounced during other-touch. This reduction correlated with ketamine-induced reductions in interoceptive awareness. The temporoparietal cortex showed higher connectivity to somatosensory cortex and insula during other- compared to self-touch. This difference was augmented by ketamine, and correlated with dissociation strength for somatosensory cortex. These results demonstrate that disrupting the self-experience through ketamine administration affects neural activity associated with self-other-differentiation in a region involved in touch perception and social cognition, especially with regard to social touch by someone else. This process may be driven by ketamine-induced effects on top-down signaling, rendering the processing of predictable self-generated and unpredictable other-generated touch more similar. These findings provide further evidence for the intricate relationship of the bodily self with the tactile sense.

## Introduction

Our sense of self is crucial for our well-being and social interactions. Self-related disturbances can be found in several psychiatric conditions, including schizophrenia [1]. Ketamine offers a pharmacological model for an altered state of self-perception as it induces short-term dissociative experiences. Here, we investigated the effects of ketamine on self-other-distinction and its neural underpinnings in the context of affective touch, using functional MRI and psychophysical measures.

The profound and multifaceted experience of selfhood is widely considered to be anchored in bodily self-awareness [2]. A coherent bodily self relies on the multimodal integration of sensory information, including interoceptive and proprioceptive signals from within our body [3, 4]. The sense of touch plays a crucial role in forming and maintaining this bodily self [5]. From early life on, touch enables us to experience bodily self-boundaries [6]. These experiences are often social in nature and involve affective touch, for example when a baby perceives caressing touch from a parent [7]. Affective touch, which is typically slow in speed and perceived as pleasant, typically involves C-tactile (CT) afferents peripherally [8] and affect-related cortical areas such as the insula centrally [9]. Interestingly, the insula is known to be involved in processing interoceptive signals, i.e. sensations from within our own body [10]. Following these observations, the CT-system has been suggested to be concerned with the establishment and maintenance of the bodily self [11]. Cortical processing of CT-mediated signals seems to differentiate between self- and non-self- generated sensations, further supporting the notion of a critical role for the social other in maintaining a functional bodily self: Activation across a broad range of regions involved in somatosensation and socio-affective processing is attenuated during self-produced touch compared to affective touch from others [12]. This raises the question: does the somatosensory system play a role in self-related dysfunctions?

Disturbances related to the sense of self can be found across several psychiatric disorders, e.g. in schizophrenia, dissociative disorders, and anorexia [13], and “Perceptions and understanding of self” has been included as a transdiagnostic Research Domain Criteria dimension [14, 15]. Such disturbances severely impact well-being and mental health: self-disorders correlate with impaired social functioning and suicidality [16]. Self-related dysfunctions are rarely addressed in currently available therapies, and they often persist even when other symptoms improve. This might be in part due to the complexity of self-related dysfunction and its co-occurrence with other symptoms.

The N-methyl-D-aspartate (NMDA) receptor antagonist ketamine is a dissociative anesthetic drug, clinically used in anesthesia and in lower doses in the treatment of depression [17, 18] and chronic pain [19, 20]. It has been suggested as a pharmacological model of self-related functional alterations, since it produces a state that resembles aspects of endogenous psychoses [21–23]. For example, ketamine administration in healthy individuals is associated with an aberrant experience of agency [24] and increased illusory body ownership [25]. If a coherent sense of self and bodily self-other-distinction are intertwined, a reduced self-other-distinction is expected in the dissociated state elicited by ketamine. However, it remains unknown if ketamine administration alters tactile self-other-distinction, and if so, through which neural mechanisms.

**Fig. 1:**
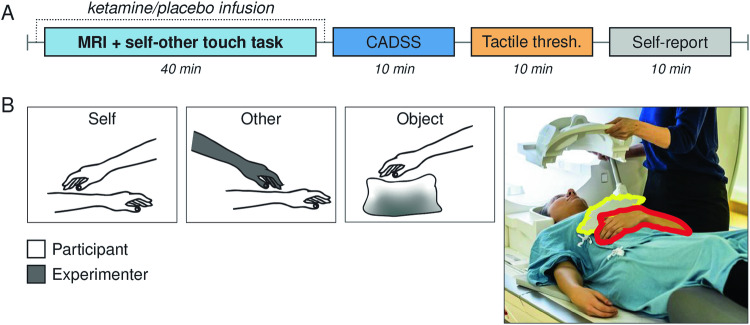
Experiment overview. **A** Timeline of the drug administration sessions. CADSS clinician administered dissociative state scale. Self-report STQ, MAIA, and AQ questionnaires. **B** Self-other-touch paradigm [12]. The position of the touched arm (red) and object (yellow) during the MRI session.

In this randomized double-blind placebo-controlled within-subject study, participants received intravenous ketamine during functional MRI, while experiencing self- or other-produced affective touch on the forearm (Fig. 1). Affective touch is operationalized as slow and gentle touch which is generally perceived as pleasant [26]. Moreover, dissociative states were assessed, and a psychophysical task was employed to determine tactile detection thresholds during self- and other-touch. Our preregistered hypotheses were that ketamine reduces the distinction of self- and non-self-generated touch, based on the known dissociative effects of ketamine at a subanesthetic dose [21]. Specifically, we hypothesized that differences in neural signatures of self-touch and other-touch [12] are smaller under ketamine than under placebo, as we have previously shown that regions involved in somatosensation and socio-affective processing differentiate between other-touch and self-touch [12]. We now predicted that these regions would differentiate less during the ketamine session when participants are expected to have dissociative experiences. Moreover, we predicted that tactile detection thresholds during self-touch are lower under ketamine, in line with notions of reduced attenuation of self-produced sensations in self-disorders [27, 28]. We further expected these changes to relate to measures of interoception and attitudes towards social touch, i.e. to alterations in how intra- and inter-subjective signals are experienced.

## Materials and methods

The study was conducted in accordance with the principles of the Declaration of Helsinki and approved by the Swedish Ethical Review Authority (2020-06515) and the Swedish Medical Products Agency. The study protocol is registered in clinical trials database EudraCT (2020-004487-25). The analysis plan was preregistered on the Open Science Framework before unblinding (https://osf.io/grud4).

### Participants

After a pre-screening during the initial phone contact, 34 potential participants were screened, of whom 31 were included. One participant dropped out during the second MRI session (visit 4), due to nausea. A total of 30 healthy participants (15 females, 15 males, mean age: 24.8 years, age range 19–39) completed the study. This sample size was preregistered and based on a power calculation for a two-phase cross-over comparison with 80% power to detect an effect size of Cohen’s D ≥ 0.6 at alpha = 0.05, i.e., a medium—large effect size. Exclusion criteria included: any clinically significant medical condition, any current clinically significant psychiatric problems including any diagnosis of alcohol or substance use disorder, history of psychotic experiences, familial history (first and second degree relatives) of psychosis or alcohol use disorder, known hypersensitivity to ketamine, use of central nervous system active medications, inability to provide a negative drug screen test, pregnancy or breastfeeding, and contraindications for MRI. Participants received 1500 Swedish kronor (approximately $150) as reimbursement. All participants provided written consent before study participation.

### Procedure

The study had a within-subject, cross-over, double-blind, randomized placebo-controlled design. The study consisted of four sessions: (1) a screening visit; (2) a baseline visit including informed consent, questionnaires (social touch questionnaire (STQ) [29], autism spectrum quotient (AQ) [30], multidimensional assessment of interoceptive awareness (MAIA) [31]), and a heartbeat detection task; and two (3 & 4) ketamine/placebo administration sessions including functional MRI (self-other-touch task), interview of dissociative experiences (Clinician-Administered Dissociative States Scale (CADSS), psychophysics (tactile threshold task), and the aforementioned questionnaires. Participants were randomized 1:1 to one of two groups which received ketamine (Ketamin Abcur 10 mg/ml, dosage 0.5 mg/kg body weight during a 40 min i.v. infusion without bolus) on either the first or second MRI session. During the other MRI session, participants received placebo (standard saline infusion). The fMRI task started approximately 20 min after the start of infusion to ensure that robust ketamine plasma concentrations were achieved. Randomization was stratified by gender. To optimize blinding, participants were informed that they may receive placebo or ketamine in any or both sessions, and that the dosage may differ between sessions. They also received basic information about the potential side-effects of ketamine, including dissociative symptoms. See supplement for a detailed description of each session and the analysis of questionnaire data.

### Experimental tasks

#### Self-other-touch paradigm

Participants performed our previously established self-other-touch paradigm (Fig. 1) [12, 32]. The task has a randomized block design and consists of three different conditions: stroking of the own left forearm (self-touch), being stroked by the experimenter (other-touch) or stroking a pillow (object-touch). To allow for stroking movements within the scanner, the left forearm was placed on the participant’s belly. For the object-touch condition, a small, sand-filled, rectangular pillow with a soft, skin-like surface was placed right above the left forearm. Participants were instructed to stroke gently, as they would touch someone they like, with their right hand. They received textual instructions regarding the upcoming block on a screen viewed through MR-compatible goggles (VisuaStim Digital; Resonance Technologies). The instructions were presented in Swedish for 3 s: “Active, please stroke your arm”; “Active, please stroke the object”; “Passive, your arm will be stroked by the experimenter.” When the text turned from white to green, the participant was stimulated or had to perform the stimulation for as long as the text was on the screen (12 s). During this condition the experimenter mimicked the motion and touched area of the participants as closely as possible. Each of the three conditions occurred 10 times and consisted of stimulation for 12 s followed by 12 s rest, resulting in a total duration of 13.5 min (Fig. 1). The female experimenter performing the strokes stood next to the scanner bore and received auditory cues on the timing of the other-touch condition via headphones. Participants met this experimenter beforehand and the same experimenter performed the touch in both sessions if logistically feasible, which was the case for 80% of the participants.

#### Touch threshold task

A previously described procedure for the touch threshold task was followed [12], using von-Frey monofilaments (Bioseb) of increasing thickness and the same three different touch conditions as during the MRI session. See supplement for a full description of the procedure and the analysis.

### Functional MRI analysis

See supplement for a detailed description of the MRI acquisition protocol and preprocessing steps. Statistical analyses were performed using the general linear model approach (SPM12). At the first-level (single-subject level), regressors of interest were the blocks of stimulation (self-touch, other-touch, and object-touch). Regressors of no interest were added for the cue phase (separately for each block) and arm movements after each active block (self-touch and object-touch; duration: 1 sec), when subjects put their arm back into a resting position. To account for movement associated variance, realignment parameters and their first temporal derivates were included as regressors of no interest, as well as a regressor censoring scans with more than 1 mm scan-to-scan movement. Contrast maps were generated for the other-touch vs. self-touch condition, as well as the self-touch vs. object-touch condition. Movement-corrected contrast maps were generated by contrasting other-touch vs. [self-touch minus object-touch].

At the second-level, a paired samples t-test was used to quantify ketamine vs. placebo effects on other-touch vs. (movement-controlled) self-touch contrast maps. For whole-brain analyses, results were corrected for multiple comparisons using the family-wise error (FWE) correction based on Gaussian random field theory at the voxel-level (as implemented in SPM and shown to be valid [33]). In addition, within four a-priori (and preregistered) regions of interest (anatomically defined), a small volume correction (SVC) was used: right posterior superior temporal gyrus, right insula, right anterior cingulate cortex, and right postcentral gyrus (functionally known as primary somatosensory cortex). Given strong evidence for involvement of the thalamus and the posterior cingulate in dissociation [34–37] exploratory (non-preregistered) analyses also assessed activation differences in these regions.

Initial analyses of a treatment (ketamine vs. placebo) effect on activation differences for self vs. other touch revealed significant voxels showing reduced activation in the cerebellum (See supplement). Control analyses showed the same effect of ketamine on activation differences for object-touch vs. other-touch, indicating that the cerebellar activation differences related to the arm movements (inherent to the self-touch and object-touch conditions), rather than touch sensations. We anticipated this issue in our preregistered analysis plan, so we used a movement-controlled analysis in the remainder of our analysis, in line with previous work [32]. For this analysis, object-touch contrast maps were subtracted from the self-touch contrast maps. Crucially, control analyses showed that ketamine did not modulate differences between self- and object-touch (paired-sample t-test on self-object contrast maps for ketamine vs. placebo, in both directions; no significant voxels on the whole-brain FWE-corrected level).

See supplement for a description of the follow-up generalized psycho-physiological interaction (gPPI) analysis [38].

## Results

### Ketamine induced a dissociative state

**Fig. 2:**
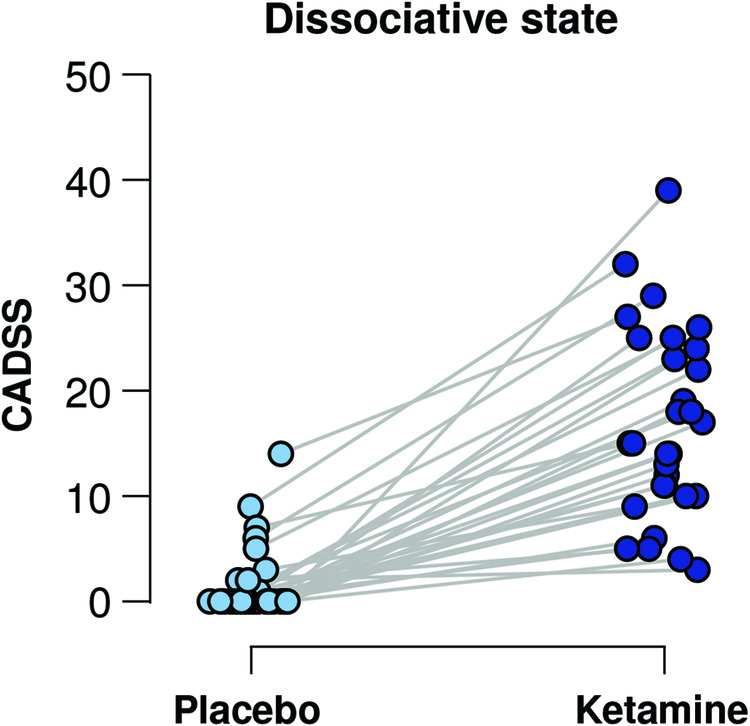
Ketamine administration induced a dissociative state. Dissociative states determined by a clinical interview (clinician administered dissociative state scale, CADSS) were higher for all participants during the ketamine session compared to the placebo session.

Participants indicated a significantly higher dissociative state after receiving ketamine compared to placebo (range increase 1–39 points, *t*(29) = 9.81, *p* < 0.001, Cohen’s *d* = 1.79, Fig. 2). See Figure S2 for difference scores on individual items and distribution across participants.

### Ketamine was associated with reduced neural distinction of self- versus other-generated touch sensations

**Fig. 3:**
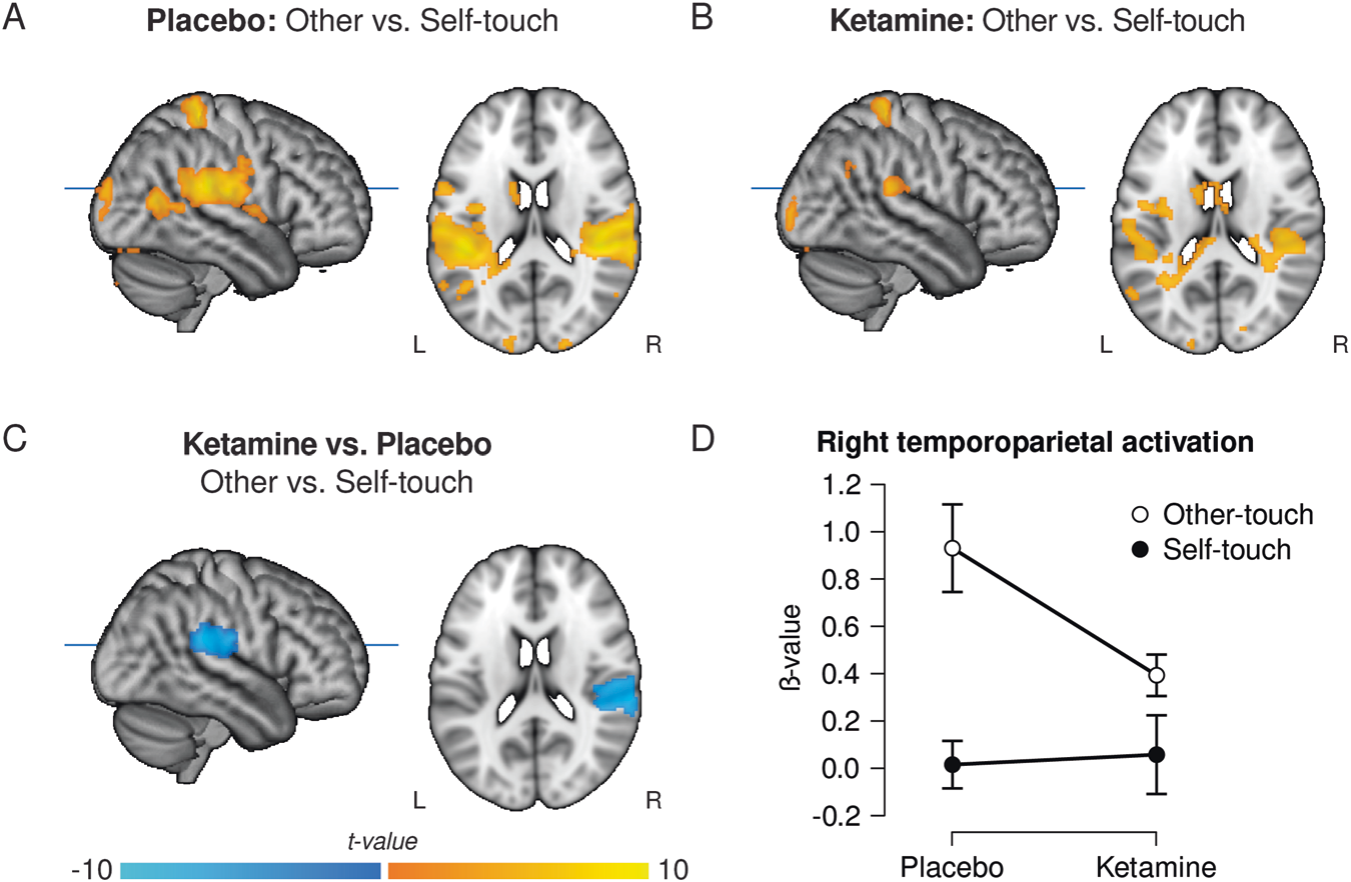
Ketamine administration was associated with a reduced distinction of self- versus other-generated touch sensations at the neural level. **A**, **B** Activation differences for other- vs. self-touch under placebo **A** and ketamine **B**. **C** Ketamine reduces self-other-differentiation in the right temporoparietal cortex. Negative values indicate a ketamine-related reduction. **D** Mean beta-values for the different treatment and touch conditions, extracted from the cluster of voxels for display purpose, showing the significant interaction depicted in **C**. All statistical maps displayed according to the neurological convention, thresholded at *p* < 0.001 uncorrected for display purpose. Axial cut-through slices are at level Z = 18 according to the MNI system.

Stronger activation for other-touch compared to self-touch during the placebo session was found in right postcentral gyrus (S1) and bilateral posterior superior temporal gyrus (pSTG)/parietal operculum, among other regions, replicating previous findings [12]. See Fig. 3A and table S1. During the ketamine session, a similar pattern was found, i.e. higher S1 and bilateral pSTG activation for other- vs. self-touch (Fig. 3B and table S2).

Comparing ketamine and placebo directly revealed that the difference in right temporo-parietal cortex (rTPC) activation for other- vs. self-produced affective touch was reduced when participants received ketamine (interaction between treatment (placebo vs. ketamine) and condition (other-touch vs. self-touch): *p*_FWE(whole brain)_ = 0.012, MNI_xyz_ = 58,−32,22; Fig. 3C; see Fig. 3D for an illustration of the nature of the interaction). The peak of this interaction effect was located on the border between the parietal operculum (or S2), pSTG, and supramarginal gyrus (SMG), and the expanded dorsally towards the anterior temporoparietal junction and ventromedially towards the posterior insula. This interaction effect overlapped with two preregistered volumes of interest: the STG (SVC *p*_FWE_ < 0.001, MNI_xyz_ = 58,−32,22) and the border of S1 (SVC *p*_FWE_ = 0.032, MNI_xyz_ = 62,−16,22). No suprathreshold voxels were found in the right ACC and right insula. Additional analyses for each condition separately (see Supplement Table S3) revealed a significant reduction in activation in the TPC under ketamine for other-touch, but not for self-touch or object-touch. See supplement for an exploratory task-based functional connectivity analysis using the rTPC as a seed region.

In sum, during ketamine administration, the neural distinction between self- and other-produced affective touch was preserved but attenuated in the right temporoparietal cortex, rendering the neural signal during other-touch more similar to the neural signal during self-touch (Fig. 3C, D). Hereafter, this interaction effect is referred to as “reduction in temporoparietal distinction”.

### Ketamine did not affect tactile detection thresholds during self- and other-generated touch

Participants showed increased touch thresholds for stimulation with von Frey filaments administered simultaneously with both the self- and other-touch condition compared to baseline in the placebo condition (Fig. S4). No evidence was found for the hypothesized reduction in tactile detection thresholds during self-touch under ketamine (*t*(28) = 0.97, *p* = 0.34), indicating that a sub-anesthetic dose of ketamine did not significantly affect basic sensing of tactile stimuli. See supplement for further analyses.

### Ketamine was associated with alterations in social touch attitudes and interoceptive awareness

Social touch (STQ) scores were lower after ketamine compared to placebo, indicating a relative increase in social touch seeking (or decrease in social touch avoidance) during the ketamine session (*t*(29) = −2.14, *p* = 0.041, Cohen’s d = 0.39). Total interoceptive awareness (MAIA) scores did not differ between ketamine and placebo sessions (*t*(29) = −0.087, *p* = 0.93). See supplement of an analysis of MAIA-subscales. Session differences for total MAIA-scores (∆-MAIA) and STQ-scores (∆-STQ) were inversely correlated (*r* = −0.41, *p* = 0.024), indicating that increases in interoceptive awareness accompanied increases in social touch seeking.

### Ketamine-induced changes in reported experiences related to changes in neural markers of self-other-distinction

**Fig. 4:**
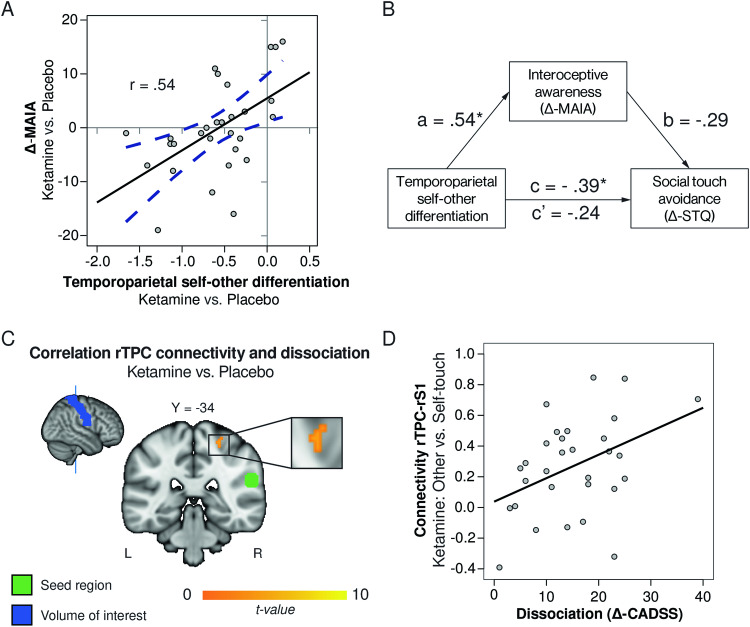
Ketamine-induced changes in experiences related to ketamine-induced changes in neural self-other-distinction. **A** Reductions in self-other-distinction correlated with reductions in interoceptive awareness (∆-MAIA). Negative values indicate a ketamine-related reduction. **B** The relationship between ketamine-induced changes in temporoparietal self-other-distinction and social touch seeking (the inverse of touch-avoidance; ∆-STQ) was mediated by ketamine-induced changes in interoceptive awareness. *p < 0.05. **C** Ketamine-induced connectivity differences (other vs. self-touch) between the right temporoparietal cortex and right S1 correlated with ketamine-induced dissociative states. Y-value refers to MNI-coordinate of cut-through slice. Maps are thresholded at *p* < 0.001 uncorrected for display purpose. **D** Visualization of the relationship between dissociation and connectivity between the temporoparietal cortex and S1, for the ketamine session. Mean beta-values for the different touch conditions are extracted from the voxels depicted in **C**.

An exploratory analysis assessed the relationships between ketamine-related changes in reported experiences and ketamine-related reductions in temporoparietal distinction. Reductions in temporoparietal distinction correlated with reductions in interoceptive awareness (∆-MAIA; *r* = 0.54, *p* = 0.002, Fig. 4A). ∆-MAIA was also associated with reductions in self-other-distinction in rInsula, rSTG, rACC, bilateral thalami and posterior cingulate cortex (see Table S6). In accordance with the negative relationship between ∆-MAIA and ∆-STQ described above, reduced temporoparietal distinction correlated negatively with increased social touch seeking tendencies (∆-STQ) (*r* = −0.39, *p* = 0.034). However, mediation analyses showed that this was an indirect relationship mediated by ∆-MAIA (indirect relationship: *B* = −0.15, bootstrapped CI [−0.35, −0.0033], *p* < 0.05, see Fig. 4B).

In an additional exploratory connectivity analysis, change in dissociation symptoms (∆-CADSS) was included as a regressor. ∆-CADSS correlated with the increase in ketamine-induced rTPC-rS1 connectivity during other- vs. self-touch (SVC *p*_FWE_ = 0.034, MNI_xyz_ = 26,−32,56, Fig. 4C, D), but not with rTPC-rIns and rTPC-rACC connectivity. See supplement for a similar analysis assessing the relationship between functional connectivity and ∆-MAIA.

## Discussion

This study investigated the effects of ketamine on self-other-distinction in the context of affective touch. Since the bodily self is established and maintained through tactile inputs (among others), we hypothesized that changing the experience of the bodily self using a sub-anesthetic dose of ketamine known to evoke dissociative experiences would be accompanied with a change in touch processing. Using an established task that shows strong neural self-other-distinction of touch under normal conditions [12], we found that this distinction was reduced in the right temporoparietal cortex (rTPC) when people experienced a mild dissociative state. Activity in this region related to changes in interoceptive awareness and its connectivity with the right S1 correlated with dissociation.

The effects of ketamine on the bodily self are of special interest as ketamine has been suggested as a model for certain aspects of schizophrenia [21, 23]. Ketamine has also shown efficacy as a treatment, especially for depression [17, 18, 39] and pain [19, 20]. However, the antidepressant effects of ketamine appear to be unrelated to the dissociative experience during ketamine administration [40, 41] and might depend on distinct underlying mechanisms [42]. The same has been suggested for the use of ketamine to manage pain [43], (but also see [44]). Our study used ketamine specifically to induce an acute altered state of self, therefore the results will be discussed mainly with a focus on their relation to the sense of self and alteration of it as seen e.g. in psychosis. Similar to other psychedelic drugs, ketamine induces an altered state of consciousness, but its effect on disembodiment is more pronounced compared to, for example, psilocybin [45]. Ketamine is a non-competitive NMDA receptor antagonist, and its pharmacological effects include disruptions of glutamatergic and dopaminergic systems [22]. Glutamatergic signaling occurs mainly via NMDA receptors at descending (top-down) connections [46]. NMDA receptor blockade leads to a reduction of top-down signaling, i.e. reduces constraints on inferences about the causes of sensory inputs [47]. Reduced (or aberrant) top-down inference will render the processing of predictable and unpredictable stimuli more similar [47]. This process was illustrated by a recent study showing that ketamine reduced activation during surprising tactile stimuli in the mouse S1 and S2 [48]. The S2 is homologous to our locus of decreased activation during the ketamine session. In the case of psychosis, it is also suggested that top-down modulation is affected [49, 50]: Predictive coding accounts assign a central role to decreased precision of prior expectations in psychosis, potentially due to increased volatility estimates [51]. These alterations in predictive coding are thought to result in aberrant associative learning and, in the long term, the formation of delusions [22, 23].

Naturally, predictive coding plays a crucial role in the distinction of self- vs. other-generated sensations, including touch. Touch perceived from others is intrinsically more unpredictable than self-generated touch [52, 53]. Consequently, the processing of self-produced sensations is attenuated and perceived as less intense than other-generated sensations. An illustration of this phenomenon is that we do not perceive self-touch as ticklish [53–55]. In the placebo condition of the current study, the stark difference in predictability of other- vs. self-generated touch was reflected in stronger activation during other-touch, a replication of our previous studies [12, 32]. In accordance with the proposed mechanism of ketamine described here, this difference in activation was reduced during the ketamine session, rendering the neural signal closer to that of self-generated touch. This suggests that the difference in predictability of other- and self-touch was reduced because of decreased top-down signaling and less constrained inferences about the causes of sensory input under ketamine.

Our results provide new information regarding two open issues in predictive coding accounts of NMDA receptor functioning in psychosis: (1) the model is opaque about the level on which the predictive coding deficits occur. We showed that ketamine affected an intermediate processing area. (2) It is unclear if the predictive processing deficits vary between sensory domains [50]. We found effects on at least two sensory (sub)domains, social touch and interoception. These are relevant for both self-experience and social interactions, which are severely impacted in psychotic experiences.

The reduction in self-other-distinction under ketamine was specific for a region in the right temporoparietal cortex, on the border of the parietal operculum, posterior superior temporal gyrus (STG), and supramarginal gyrus (SMG). The parietal operculum is the anatomical site of the secondary somatosensory cortex (S2) [56] and is consistently involved in tactile self-other-distinction [12, 53, 55]. The S2 is one of the main output regions of the primary sensory cortex (S1), and is involved in a broad range of somatosensory functions, including (affective) body perception [57]. Lesion studies suggest that the S2 (together with the insula) is critical for the conscious perception of touch [58]. Both the posterior STG and SMG are consistently involved in social cognition [59]. This appears to be in line with their location, in between a unimodal processing area, S1, and a transmodal/abstract area, the temporoparietal junction (TPJ) [59, 60]. The TPJ plays a role in cognitive self-other-distinction, whereas affective self-other-distinction involves the SMG [61]. Taken together, ketamine affected tactile processing in an intermediate processing area located between the S1, responsible for processing the primary tactile input, and higher-order processing areas, including (1) the insula, which plays an important role in the conscious perception of touch and its integration with interoceptive information from the body [10], and (2) the TPJ, involved in social cognition [59]. Underlining this central role in touch processing, rTPC showed in our study an increased task-based connectivity to the right S1, right insula, and the thalamus during other-touch. rTPC-rS1 connectivity strength was positively correlated to dissociation symptoms under ketamine. These findings fit with previous reports that link (thalamic) hyperconnectivity with both ketamine and dissociation symptoms [34, 35, 62]. This is thought to reflect an overabundance of sensory information [35] and disruptions in the integration thereof [63]. The fact that ketamine induced increased connectivity with the rTPC but reduced its activation may be a result of a lack of specificity (or precision) of the incoming sensory signal.

The effect of ketamine on neural self-other-distinction paralleled a shift in interoceptive awareness, which was in turn associated with social touch seeking. Understanding others involves the engagement of brain and bodily functions primarily used to assess our own state [61]. However, for this mechanism to work adequately, it is important that we are able to distinguish ourselves from others [61]. Here, we show that blunted interoception is associated with blunted neural self-other-distinction, in line with the notion that our internal model of ourselves is highly dependent on interoceptive processing [3]. This relationship is also in line with the suggestion that the C-tactile-system is concerned with the establishment and maintenance of the bodily self [11] and the consistently reported involvement of the insula in both social touch perception and interoception [9, 10]. Increased touch seeking might be interpreted as a direct reaction to perceiving the own body and its boundaries less clearly under ketamine, in line with the proposed role of social touch in strengthening (and re-establishing) these bodily self-boundaries.

Ketamine significantly reduced activation in the cerebellum during self- vs. other- and object- vs. other-touch, indicating a potential effect of ketamine on the neural processes underlying action generation. This interpretation is corroborated by additional analyses showing reduced cerebellar activation during ketamine for both object- and self-touch separately. However, no evidence was found within this sample for a specific effect of self-oriented action (i.e. self- vs. object-touch). We did not have any measurement of touch parameters during the MRI scan, therefore, we cannot evaluate changes in the touching performance. However, even if the touching during self- and object-touch condition would have differed slightly between sessions, it probably would not have altered our main finding on the sensory aspects of self-other distinction, because the ketamine-related rTPC activity differences were strongest in the other-touch condition, which lacks an action generation component.

In contrast to the effects of ketamine on the neural level, we did not find any significant differences between tactile detection thresholds for self- and other-touch during the ketamine and placebo sessions. This could indicate that a subanesthetic dose of ketamine does not affect basic tactile discrimination in general, which fits with the fact that the neural effects did not occur in the primary somatosensory area. However, the results should be interpreted with caution, as we did not replicate earlier findings on differential thresholds during self-touch and other-touch in the placebo condition, suggesting that our manipulation was not completely successful. Moreover, the tactile detection thresholds were assessed approximately 15 min after administration ended, so the ketamine effects may already have been wearing off.

Participants in the current study had an accurate intuition about whether they had received ketamine during a session or not—a common challenge in placebo-controlled psychedelic studies. Although there is no obvious mechanism by which this awareness may have influenced our main result, it would be valuable to compare our results with other psychoactive drugs. Another limitation of the current study is that it did not evaluate the phenomenology of the actual touch, for example by evaluating differences in subjective experiences like perceived intensity of pleasantness. While of interest, such a measure was not implemented due to methodological concerns, i.e. the risk of drawing or altering the attention to the touch, inducing expectations about the task’s purpose, and altering touching behavior during the task. Our results may also have been influenced by potential differences in the subjective experience of touch from a relative stranger (i.e. the experimenter). Furthermore, no baseline dissociation scores were collected, but the low CADSS scores during the placebo session suggest low baseline scores (as one would expect the placebo effect to increase not decrease a baseline level of dissociation). The absence of plasma measurements of ketamine and its metabolites is another limitation. Such measurements would have been difficult to acquire in the MR setting and could have interfered with the touch task, but would have been valuable for evaluating individual differences in the pharmacokinetics/dynamics.

## Conclusion

This study demonstrated that pharmacologically manipulating the experience of the bodily self is accompanied by a change in neural processing of affective touch. During ketamine administration, self-other-distinction was reduced in a region associated with touch perception and social cognition. This process may be driven by a ketamine-induced reduction in top-down signaling, rendering the processing of predictable self-generated and unpredictable other-generated touch more similar. Our findings provide further evidence for the intricate relationship of the bodily self with social touch.

## Supplementary information

Supplemental material

Table S4

Table S5

CONSORT Flow Diagram

## Data Availability

Unthresholded activation maps are available at NeuroVault, https://identifiers.org/neurovault.collection:17403. The rest of the datasets generated during and analysed during the current study are not publicly available due restrictions from the local ethical committee, but are available from the corresponding author on reasonable request.
