# Publisher Correction: Ketamine reduces the neural distinction between self- and other-produced affective touch: a randomized double-blind placebo-controlled study

**DOI:** 10.1038/s41386-024-01916-0

**Published:** 2024-07-22

**Authors:** Reinoud Kaldewaij, Paula C. Salamone, Adam Enmalm, Lars Östman, Michal Pietrzak, Hanna Karlsson, Andreas Löfberg, Emelie Gauffin, Martin Samuelsson, Sarah Gustavson, Andrea J. Capusan, Håkan Olausson, Markus Heilig, Rebecca Boehme

**Affiliations:** 1 Center for Social and Affective Neuroscience, Linköping University, Linköping, Sweden; 2 Center for Medical Image Science and Visualization, Linköping University, Linköping, Sweden

**Keywords:** Sensorimotor processing, Perception, Social neuroscience, Human behaviour

Correction to: *Neuropsychopharmacology* 10.1038/s41386-024-01906-2, published online 25 June 2024

Due to a typesetting mistake, the funding information was incomplete. The Funding section has been corrected from “Open access funding provided by Linköping University” to “Open access funding provided by Linköping University. This research was supported by a Svenska Vetenskapsrådet (VR) grant (2019-01873) and Åke Wiberg Stiftelse grant (M19-0369) awarded to RB, and an NWO-Rubicon grant (019.211SG.005) awarded to RK.” The original article has been corrected.

