# Supplementary material for: Ketamine reduces the neural distinction between self- and other-produced affective touch: a randomized double-blind placebo-controlled study
Source: Neuropsychopharmacology. 2024 Jun 25;49(11):1767–74. doi: 10.1038/s41386-024-01906-2 (PMC11399133; doi:10.1038/s41386-024-01906-2)
Supplement: Supplementary file 1 — Supplemental material [file 41386_2024_1906_MOESM1_ESM.pdf]

Supplemental information for "*Ketamine reduces the neural distinction between self- and other-produced affective touch - a randomized double-blind placebo-controlled study*"

Supplementary methods

Procedure

Participants were included between 2021-06-30 and 2022-11-09 at the Center for Social and Affective Neuroscience, Linköping University Hospital, Linköping, Sweden. All data was collected at this location. The 4 visits (screening, inclusion, 2 test sessions) took place within a timespan between 9 and 57 days (median: 30.5 days). Time between both test sessions (ketamine/placebo administration) was between 6 and 35 days (median: 14 days).

Visit 1: Screening

The screening session included: an interview by a research nurse about their general health status and concomitant medications; blood and urine samples for general lab tests, drug tests and a pregnancy test (hCG) for women; blood pressure and heart rate assessment; self-report questionnaires on mental health (Modified Mini Screen, Alcohol Use Disorder Identification Test (AUDIT) and Drug Use Disorder Identification Test (DUDIT)) [1–3]; and a questionnaire to assess eligibility for the MRI scan. Six out of 30 participants were left-handed. Of the 4 out of 30 participants with a score >0 on the DUDIT (but below the cut-off of 4) none reported ketamine use, and no other participants had any previous experience of any illicit substance use.

Visit 2: Informed consent and baseline measurements

During the second session, the participants met with a physician to ask questions and sign the informed consent. Subsequently, participants filled out the validated Swedish translations of the following questionnaires: social touch questionnaire (STQ) [4], autism spectrum quotient (AQ) [5], multidimensional assessment of interoceptive awareness (MAIA) [6]. The STQ measures daily life social touch attitudes; the AQ quantifies individual autistic traits; the MAIA evaluates the participant's awareness of interoceptive signals from within their own body in everyday situations. Individual autistic traits were assessed because of the

associated alterations in both sensory (tactile) processing and social cognition. Afterwards, participants performed a heartbeat detection task [7] that will be reported separately. Subsequently, they visited a training scanner (PST MR Simulator System, BlindSight GmbH, Schlitz, Germany) to familiarize themselves with the scanner environment and to practice performing the functional MRI task – including instructed touch on the radial area of the forearm - while remaining as still as possible, using a head motion tracking and real-time feedback system (MoTrak, Psychology Software Tools, Inc., USA).

Randomization

Randomization and blinding were independent of study personnel, by Forum Östergötland, the clinical trials service of Linköping University Hospital, and the hospital pharmacy. Because the within-subject design of this study, only the sequence of sessions (ketamine followed by placebo, vs. placebo followed by ketamine) was randomized, stratified for sex, using pre-generated random number lists. The pharmacy prepared the infusion bags and added a blinded label before distribution to the site. All study personnel remained blinded throughout the whole data collection phase of the study.

Visit 3 & 4: Ketamine/placebo administration, MRI and psychophysics

After checking for new medications, participants' heart rate and blood pressure were measured. A urine test was collected for a drug screening and a pregnancy (human chorionic gonadotropin) test for women. Subsequently, participants received ketamine or placebo intravenously during a 40 min MRI scan. The dosage was 0.5 mg/kg body weight during a 40 min i.v. infusion without bolus – which is a sub-anesthetic dosage, comparable to the dose-range used in depression-treatment [8]. This is estimated to achieve a plasma concentration of appr. 0.2 µg/ml. The protocol reliably results in emergence of dissociative effects within 10 min of infusion start. This is consistent with observations that dissociative effects of ketamine require plasma concentrations ≥0.05 µg/ml [9]. Heartrate, breathing, and overall wellbeing were uninterruptedly monitored by a research nurse in the scanner room. Immediately after the start of the infusion and before starting the scanning procedure, participants were

monitored for 5 minutes while lying on the MRI scanner bed outside the scanner bore, to ensure the participants felt comfortable and did not develop any early adverse symptoms. The MRI session consisted of anatomical scans (12 min) and the self-other-touch task (13.5 min), both of which are described in more detail below, amounting to approximately 40 min including preparation times and intermediate communication. The fMRI task started 20 min after the start of infusion. Directly after the scanning procedure, ketamine/placebo administration was terminated. Participants were then interviewed by a research nurse about their dissociative experiences using the Clinician-Administered Dissociative States Scale (CADSS). This was followed by the tactile detection threshold test and the auditory detection threshold test (see below). Afterwards, participants filled out the STQ, AQ, and MAIA (see visit 2).

At the end of each session, participants were asked to indicate on a visual analogue scale (VAS) if they think they received ketamine or placebo. This question was included to have the option to correct for potential individual differences in beliefs about the administered drug. However, the variance in this self-report measure was very low (Perceived likelihood of having received placebo during the placebo session: median 98%, range 22-100%; perceived likelihood of having received placebo during the ketamine session: median 0%, range 0-34%).

Materials and apparatus

MRI

Magnetic resonance imaging (MRI) data was acquired with a 3.0 Tesla Siemens scanner (Prisma; Siemens) with a 64 channel head coil. Anatomical images were obtained using a T1-weighted scan (repetition time=2300 ms; echo time=2.36 ms; flip angle=8°; field of view=288 x 250 mm^2; voxel resolution=0.87 x 0.87 x 0.90 mm^3). Task-based imaging data were collected using an echo planar imaging (EPI) (repetition time: 1030 ms; echo time: 30 ms; slice thickness: 3 mm; matrix size: 64 × 64; field of view: 192 × 192 mm^2; in-plane voxel resolution: 3 mm^2; flip angle: 63°).

Touch threshold task

A previously described procedure for the touch threshold task was followed (12) Participants were seated at a table, resting their left forearm on the table. As a baseline measurement, they were asked to close their eyes and indicate when they perceived stimulation with von-Frey monofilaments (Bioseb) of increasing thickness (indentation forces 0.08–78.5 mN). Subsequently, participants underwent the same three different touch conditions as during the MRI session (e.g. self-touch, other-touch, object-touch; order randomized across subjects), while blinded and receiving simultaneous stimulation with a von-Frey-filament on the left arm. For all conditions, the perceptual threshold was defined as the weakest filament (in mN) that was detected in at least 5 out of 10 trials.

Questionnaires & Touch-threshold task analysis

Psychophysical and questionnaire data were analyzed using SPSS analysis software. Behavioral data was examined for homogeneity of variance, and if necessary, log-transformed to meet this criterion. This was the case for the tactile discrimination task. For the CADSS, one item was missing for two participants. The value of this missing item score was interpolated by calculating a rounded average score of all other items for that specific participant. The threshold during self-touch for ketamine and placebo sessions was analyzed with a paired-sample t-test. The same approach was used to assess session differences for CADSS, MAIA, STQ, and AQ. Repeated measures ANOVA with treatment and condition as within-subjects factors were used for exploratory analyses of the tactile detection thresholds. A probability level of 0.05 or lower was considered significant.

Functional MRI: preprocessing

MRI data was analyzed using SPM12 (SPM, Wellcome Department of Imaging Neuroscience, London, UK; http://www.fil.ion.ucl.ac.uk/spm) in Matlab (MathWorks, Natick, MA, USA). The preprocessing consisted of: motion correction; co-registration of the mean EPI and the anatomical (T1) image; segmentation of the T1 image and spatial normalization to the

Montreal Neurological Institute (MNI) T1 template; application of the normalization parameters to all EPI volumes; spatial smoothing of all images with an isotropic Gaussian kernel of 6-mm full width at half-maximum.

Functional MRI: generalized psycho-physiological interaction analysis

As a follow-up analysis, we conducted a generalized psycho-physiological interaction (gPPI) analysis 29 with the different task conditions as separate regressors. The seed region was a 8 mm radius sphere around the peak voxel of the obtained ketamine by task interaction effect (58,-32,22; see Results). At the second-level, a paired samples t-test was used to quantify ketamine vs. placebo effects on other-touch vs. (movement-controlled) self-touch contrast maps from the first level gPPI analysis. In two additional analyses, Δ-CADSS and Δ-MAIA scores were added as regressors to assess their interactions with task and treatment effects.

Supplementary Results

Ketamine effects on other vs. self-touch without controlling for movement

Initial analyses of a treatment (ketamine vs. placebo) effect on activation differences for other- vs. self-touch revealed significant voxels showing reduced activation in the cerebellum (Figure S1). Control analyses showed a similar effect of ketamine on activation differences in the cerebellum for other- vs. object-touch (T = 6.62, peak-level pFWE = .013, MNI_{xyz} =24,-46,-22). Ketamine did not modulate differences between self- and object-touch (no significant voxels on the whole-brain FWE-corrected level). See below for ketamine effects on the individual conditions.

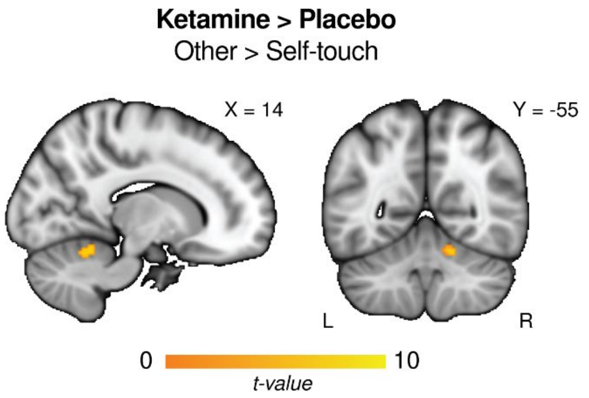

Figure S1. Ketamine effects on other vs. self-touch without controlling for movement. T = 6.51, peak-level pFWE = .010, MNI_{xyz} =16,-50,-20.

CADSS item scores

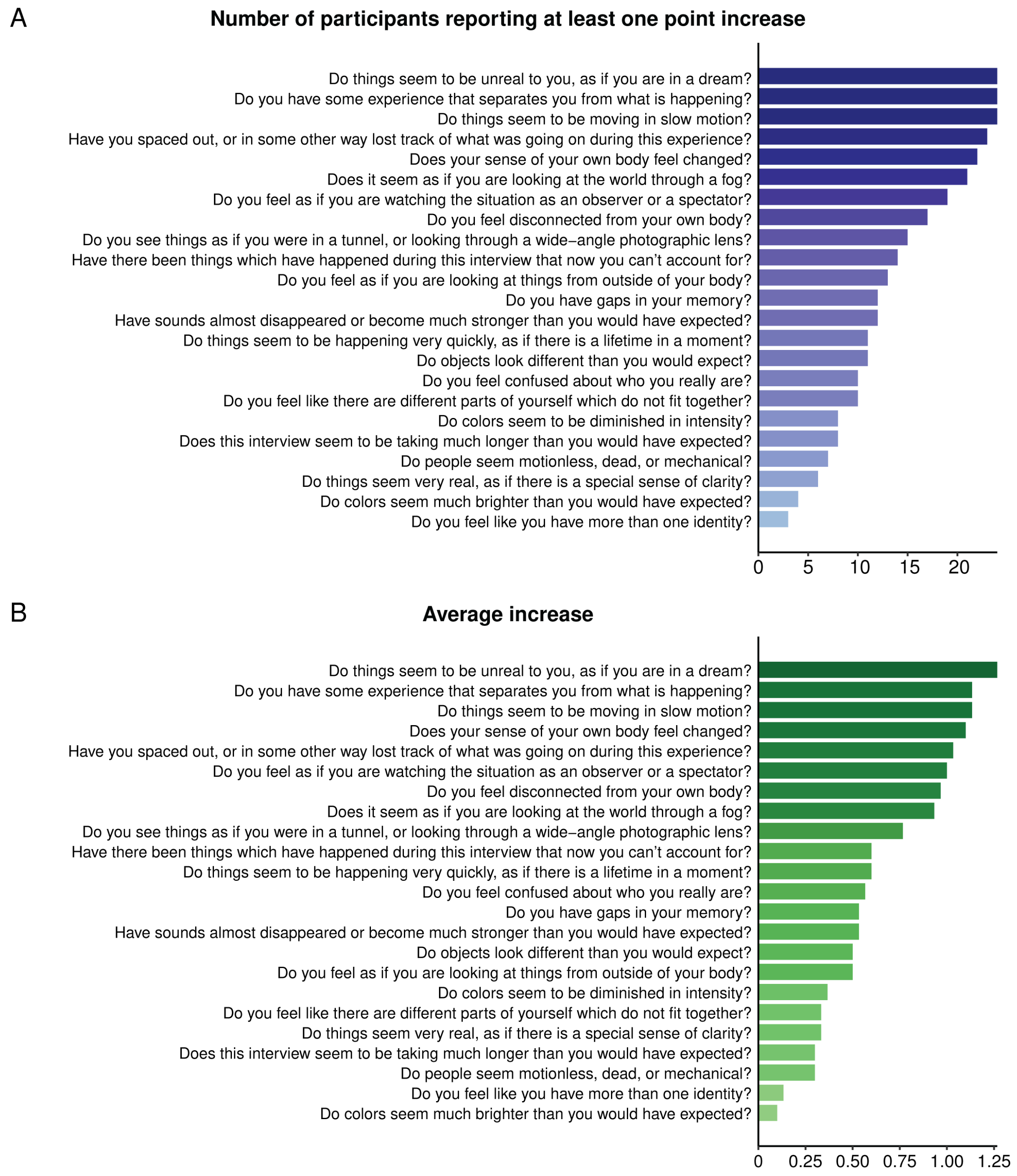

Figure S2. CADSS item scores. See Figure 2A in the main manuscript. **A.** Number of participants reporting at least one point increase per item**. B.** Average increase for each item.

Task condition effects for placebo and ketamine sessions separately

Region	*k*	L/R	x	y	z	*t*	*p(FWE)*
Operculum	401	L	-44	-38	24	13.57	< .001
Cuneus	280	L	-12	-92	0	13.5	< .001
Cuneus	273	R	12	-90	2	11.24	< .001
			14	-82	-2	9.18	< .001
			14	-72	-4	6.13	.033
Insula	569	R	44	-32	22	11.15	< .001
			54	-32	20	9.02	< .001
			60	-18	18	7.31	.002
Postcentral gyrus	57	R	22	-38	64	8.03	< .001
			24	-40	72	6.64	.010
			30	-38	62	6.44	.016
Supramarginal gyrus	80	L	-60	-22	22	7.74	.001
			-58	-20	14	6.69	.009
Fusiform gyrus	10	L	-28	-78	-8	6.86	.006
Insula	8	L	-38	-8	14	6.57	.012
Insula	14	L	-34	-22	8	6.51	.014
Heschl gyrus	6	R	56	-64	12	6.5	.014
Inferior parietal lobe	15	L	-58	-18	40	6.44	.016
Lingual gyrus	5	L	-10	-78	-8	6.4	.018
Fusiform gyrus	18	L	-34	-22	-12	6.37	.019
			-40	-28	-12	6.09	.035
Operculum	2	L	-46	-8	6	6	.043
Supramarginal gyrus	1	L	-58	-34	24	5.98	.045
Cerebellum	1	L	-18	-74	-16	5.96	.047

Table S1. Significant peak voxels for the contrast other vs. self (corrected for movement) for the placebo session only. $p < .05$, FWE-corrected.

Region	*k*	L/R	x	y	z	*t*	*p(FWE)*
Lingual gyrus	265	L	-10	-90	0	11.50	< .001
			-12	-82	-8	6.82	.006
Cuneus	312	R	12	-88	4	10.13	< .001
			12	-82	-2	9.55	< .001
Fusiform gyrus	88	L	-42	-6	-24	8.44	< .001
Superior temporal gyrus/Supramarginal gyrus	38	R	44	-32	22	7.15	.003
Superior temporal gyrus	26	L	-44	-36	24	6.94	.005
Hippocampus	63	L	-32	-32	-6	6.72	.008
			-26	-16	-14	6.49	.013
			-32	-22	-12	6.39	.016
Postcentral gyrus	11	R	24	-38	62	6.46	.014
	4	L	-16	-96	10	6.31	.019
Calcarine cortex	3	R	2	-94	-2	6.29	.020
Middle temporal gyrus	4	L	-52	-14	-24	6.27	.021

Table S2. Significant peak voxels for the contrast other vs. self (corrected for movement) for the ketamine session only. $p < .05$ FWE-corrected.

Ketamine effects on neural activation during each condition separately

The effects of ketamine on the three conditions (other, self, and object) were also assesed separately (see table below). For other-touch, a negative effect of ketamine was found on right temporoparietal cortex activity (similar to the other vs, self (movement-corrected) contrast). For both self-touch and object-touch, we found very similar negative effects of ketamine on activation in the right cerebellum, consistent with the above-mentioned ketamine effects on other vs. self and other vs. object contrasts. No positive effects (i.e. increased activity) of ketamine were found.

Contrast	Region	k	L/R	x	y	z	T	*p*(FWE)
Other>Self(mov corr.)*	Temporoparietal cortex	13	R	58	-32	22	6.59	.012
Other	Temporoparietal cortex	164	R	44	-32	22	7.94	.001
	Temporoparietal cortex	16	L	-44	-32	22	7.22	.012
Self	Cerebellum	24	R	16	-52	-18	6.63	.008
Object	Cerebellum	31	R	22	-48	-20	6.85	.008

Table S3. Effects of ketamine. Placebo vs. ketamine; effects reflect a relative reduction after ketamine administration. *Main interaction effect as reported in the manuscript.

Ketamine administration augments existing functional connectivity patterns with the temporoparietal cortex

To assess the role of the rTPC during self-other-distinction and dissociation further, we ran an exploratory task-based functional connectivity analysis (generalized psychophysical interaction analysis[10]), using the rTPC as a seed region. During the placebo-session, rTPC-rS1 and rTPC-rIns functional connectivity (among other regions, see table S4) was higher for other-touch compared to self-touch (whole-brain corrected) (Figure S3A). A similar pattern was found during ketamine-administration (Figure S3B and table S5). Comparing ketamine- and placebo-sessions directly revealed no significant differences in rTPC connectivity at the whole-brain level. However, small-volume-corrected analyses for two preregistered a-priori defined regions of interest, the rS1 and rInsula, showed that the higher rTPC-rS1 and rTPC-rIns connectivity during other-touch vs. self-touch was augmented during the ketamine session (rS1: p_{FWE}=.018, MNI_{xyz}=18,-32,72; rInsula: p_{FWE}=.029, MNI_{xyz}=46,0,6; no significant connectivity differences for rACC; rSTG volume of interest omitted because of seed region overlap). Given strong evidence for involvement of thalamic connectivity and the posterior cingulate in dissociation [11–14], exploratory analyses also assessed connectivity between these regions and the rTPC. These analyses indeed revealed increased connectivity during ketamine between the right temporoparietal cortex (rTPC, the seed region), and both the right (p_{FWE} = .042, MNI_{xyz}=10,-6,4) and left thalamus (p_{FWE} = .006, MNI_{xyz}= -10,-24,0) when comparing other vs. self-touch. No evidence for ketamine-related differences in rTPC-posterior cingulate cortex connectivity was found.

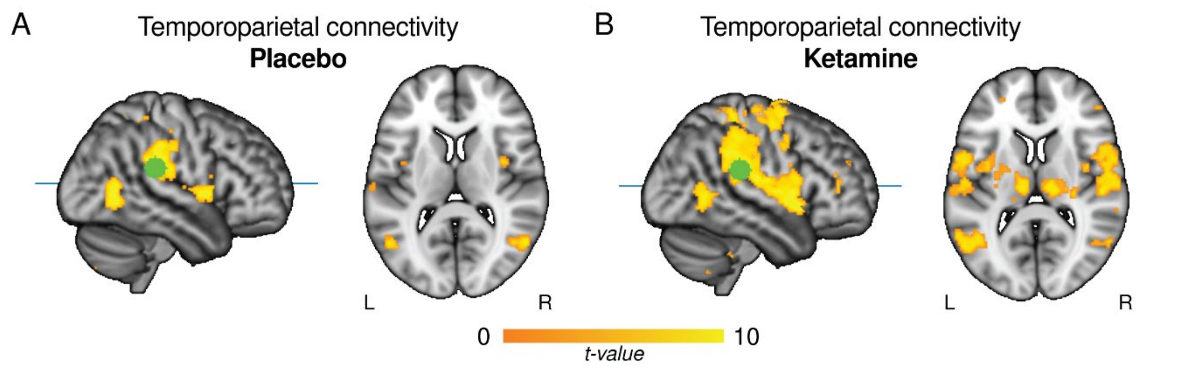

Figure S3. Ketamine augments existing functional connectivity patterns with the temporoparietal cortex (rTPC). A-B. Temporoparietal connectivity differences for other- vs. self-touch under placebo (A) and ketamine (B). $p<.05$ FWE-corrected. Axial cut-through slices are at $Z_{MNI}=11$. Seed region (rTPC) indicated in green.

Connectivity effects for placebo and ketamine sessions separately

[Table S4 is provided as a separate excel file]

Table S4. Peak voxels showing significant connectivity (with right temporoparietal seed) for the contrast other vs. self (corrected for movement) for the placebo session only. $p < .05$, FWE-corrected.

[Table S5 is provided as a separate excel file]

Table S5. Peak voxels showing significant connectivity (right temporoparietal seed) for the contrast other vs. self (corrected for movement) for the ketamine session only. $p < .05$, FWE-corrected.

Tactile threshold task

A repeated measures ANOVA with factors treatment (ketamine vs. placebo) and task condition (baseline, self-touch, other-touch, object-touch) did not show any significant treatment effect ($F(1,28)=0.47$, $p=.50$) or a significant treatment * task condition interaction ($F(3,84)=1.27$, $p=.29$), but did show a significant effect of task condition ($F(3,84)=144.00$, $p<.001$). Bonferroni-corrected post-hoc pairwise comparisons showed that the effect of condition was driven by higher thresholds during both self- and other-generated touch compared to both object-touch and baseline (all $p<.001$; Figure S4).

There were no significant relationships between ketamine-induced dissociation symptoms (Δ-CADDS), Δ-MAIA or Δ-STQ and psychophysical self-other-distinction, or any relationships between the and psychophysical markers of self-other-distinction.

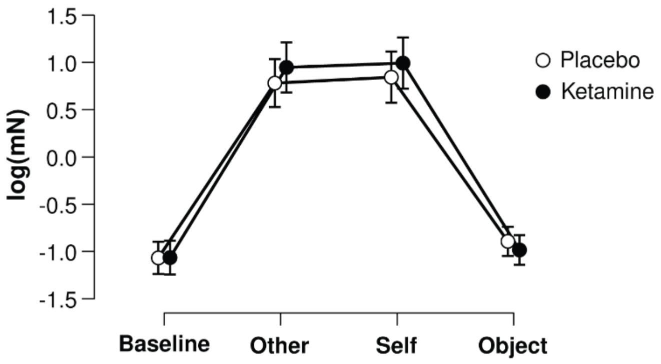

Figure S4. Tactile detection thresholds. Y-axis depicts log-transformed forces in millinewton.

AQ and MAIA (interoceptive awareness) subscale scores

Autism quotient (AQ) scores did not significantly differ between sessions (Wilcoxon signed-rank; z=-0.55, p=.58). The MAIA subscales "not-worrying" and "trusting" were reduced during the ketamine session (Wilcoxon signed rank tests; z = 2.50, p = .013; z = 2.14, p = .032; respectively). This indicates an increased "tendency to worry or experience emotional distress with sensations of pain or discomfort" and a decreased "experience of one's body as safe and trustworthy" during the ketamine session. Note, however, that the MAIA has eight subscales and that these effects do not survive correcting for multiple comparisons.

Relationship between Δ-MAIA score and ketamine-induced changes in neural activation during self-other-distinction

Region	*k*	L/R	x	y	z	*t*	*p(FWE)*
Insula[1]	5	R	42	2	-12	4.90	.009
	1	R	40	0	-4	4.33	.033
Superior temporal gyrus[1]	1	R	44	0	-14	4.95	.013
	3	R	46	-48	20	4.55	.031
Anterior cingulate cortex[1]	2	R	10	40	-2	4.05	.042
	2	R	2	32	24	4.04	.044
Thalamus	21	L	-16	-12	2	4.8	.006
	3	R	16	-24	10	4.19	.023
	3	R	18	-30	0	4.02	.033
Posterior cingulate cortex	4	R	6	-40	42	3.55	.045

Table S6. Small-volume-corrected analyses for the contrast ketamine (other>self) > placebo (other>self) covarying with Δ-MAIA. Voxels in the preregistered regions of interest right ACC, right STG, right insula, thalamus and posterior cingulate cortex show a positive relationship between increases in interoceptive awareness and self-other-distinction, suggesting that increases in interoceptive awareness under ketamine are related to (more) intact self-other-distinction. This correlation was not significant for our fourth preregistered region of interest, the right postcentral gyrus (S1). $p < .05$, FWE-corrected. [1] Preregistered region of interest

Relationship between Δ-MAIA score and ketamine-induced changes in functional connectivity during self-other-distinction

In this additional connectivity analysis, ketamine-induced changes in interoceptive awareness (Δ-MAIA) were included as a predictor. Δ-MAIA correlated with the increase in ketamine-induced connectivity during other- vs. self-touch between the rTPC and right insula (peak pFWE = .015, MNIxyz = 40,-2,0, small-volume corrected).

Effects of sex and alcohol consumption on dissociation and neural activation during self-other distinction.

Exploratory analyses were run to examine potential effects of both sex and alcohol consumption on the main outcomes of this study. CADSS difference scores (ketamine-placebo) were not significantly different for males and females ($t(28) = 0.085$, $p = .93$). Similarly, there was no effect of sex on the mean extracted beta-values from the main activation cluster (i.e. the interaction effect in the temporoparietal cortex, see figure 3C) ($t(28) = 0.20$, $p = .85$). Alcohol

consumption (AUDIT) scores did not correlate significantly with the ketamine-induced shift in dissociation (delta-CADSS; r = -.022, p = .91) or reduction in self-other differentiation in the right temporoparietal cortex (mean beta values from the activation cluster; r = -.14, p = .47).

References

1. Berman AH, Bergman H, Palmstierna T, Schlyter F. Evaluation of the Drug Use Disorders Identification Test (DUDIT) in criminal justice and detoxification settings and in a Swedish population sample. Eur Addict Res. 2005;11:22–31.
2. Saunders JB, Aasland OG, Babor TF, Fuente JRDELA, Grant M. Development of the Alcohol Use Disorders Identification Test (AUDIT): WHO Collaborative Project on Early Detection of Persons with Harmful Alcohol Consumption — II. 1993:791–804.
3. Alexander MJ, Haugland G, Lin SP, Bertollo DN, Mccorry FA. Mental Health Screening in Addiction , Corrections and Social Service Settings : Validating the MMS. 2008:105–119.
4. Wilhelm FH, Kochar AS, Roth WT, Gross JJ. Social anxiety and response to touch: Incongruence between self-evaluative and physiological reactions. Biol Psychol. 2001;58:181–202.
5. Baron-cohen S, Wheelwright S, Skinner R, Martin J, Clubley E. The Autism-Spectrum Quotient (AQ): Evidence from Asperger Syndrome / High-Functioning Autism , Males and Females , Scientists and Mathematicians. 2001;31.
6. Mehling WE, Price C, Daubenmier JJ, Acree M, Bartmess E, Stewart A. The Multidimensional Assessment of Interoceptive Awareness (MAIA). 2012;7.
7. Salamone PC, Legaz A, Sedeño L, Moguilner S, Fraile-Vazquez M, Campo CG, et al. Interoception primes emotional processing: Multimodal evidence from neurodegeneration. J Neurosci. 2021;41:4276–4292.
8. Zarate CA, Singh JB, Carlson PJ, Brutsche NE, Ameli R, Luckenbaugh DA, et al. A randomized trial of an N-methyl-D-aspartate antagonist in treatment-resistant major depression. Arch Gen Psychiatry. 2006;63:856–864.
9. Zanos P, Moaddel R, Morris PJ, Riggs LM, Highland JN, Georgiou P, et al. Ketamine and ketamine metabolite pharmacology: Insights into therapeutic mechanisms. Pharmacol Rev. 2018;70:621–660.
10. McLaren DG, Ries ML, Xu G, Johnson SC. A generalized form of context-dependent psychophysiological interactions (gPPI): A comparison to standard approaches. Neuroimage. 2012;61:1277–1286.
11. Onofrj M, Russo M, Delli Pizzi S, De Gregorio D, Inserra A, Gobbi G, et al. The central role of the Thalamus in psychosis, lessons from neurodegenerative diseases and psychedelics. Transl Psychiatry. 2023;13.
12. Abram S V., Roach BJ, Fryer SL, Calhoun VD, Preda A, van Erp TGM, et al. Validation of ketamine as a pharmacological model of thalamic dysconnectivity across the illness course of schizophrenia. Mol Psychiatry. 2022;27:2448–2456.
13. Vesuna S, Kauvar I V., Richman E, Gore F, Oskotsky T, Sava-Segal C, et al. Deep posteromedial cortical rhythm in dissociation. Nature. 2020;586:87–94.
14. Parvizi J, Braga RM, Kucyi A, Veit MJ, Pinheiro-Chagas P, Perry C, et al. Altered sense of self during seizures in the posteromedial cortex. Proc Natl Acad Sci U S A. 2021;118:1–9.
