# Supplementary material for: Ketamine reduces the neural distinction between self- and other-produced affective touch: a randomized double-blind placebo-controlled study
Source: Neuropsychopharmacology. 2024 Jun 25;49(11):1767–74. doi: 10.1038/s41386-024-01906-2 (PMC11399133; doi:10.1038/s41386-024-01906-2)
Supplement: Supplementary file 4 — CONSORT Flow Diagram [file 41386_2024_1906_MOESM4_ESM.docx]

Excluded (n= 3)

- Not meeting inclusion criteria (n= 3)

Analysed (n= 16)

Analysed (n= 14)

- Excluded due to nausea during second MRI scan (n= 1)

## Analysis

Lost to follow-up (n= 0)

Lost to follow-up (n= 0)

Saline followed by ketamine (n= 15)

- Received allocated intervention (n= 15)

- Did not receive allocated intervention (n= 0)

## Follow-Up

Ketamine followed by saline (n= 16)

- Received allocated intervention (n= 16)

- Did not receive allocated intervention (n= 0)

## Allocation

Randomized (n= 31)

Assessed for eligibility (n= 34)
